# Bovine ringworm - Detection of *Trichophyton verrucosum* by SYBR-Green real-time PCR

**DOI:** 10.1016/j.mmcr.2023.01.002

**Published:** 2023-01-31

**Authors:** Andréia Spanamberg, Ana Paula Ravazzolo, Ricardo Araujo, Natália Franceschi, Laerte Ferreiro

**Affiliations:** a Setor de Micologia Veterinária, Departamento de Patologia Clínica Veterinária, Universidade Federal do Rio Grande do Sul (UFRGS), Porto Alegre, RS, Brazil; b Programa de Pós-graduação em Ciências Veterinárias (PPGCV), UFRGS, Porto Alegre, RS, Brazil; c Laboratório de Imunologia e Biologia Molecular, Faculdade de Veterinária, UFRGS, Brazil; d i3S, Instituto de Investigação e Inovação em Saúde, Porto, Portugal; e INEB – Instituto Nacional de Engenharia Biomédica, Universidade do Porto, Portugal

**Keywords:** *Trichophyton verrucosum*, Dermatophytes, Hair, Cattle, Ringworm

## Abstract

*Trichophyton verrucosum* is the most commonly dermatophyte involved in cattle ringworm. This work reported a case of bovine dermatophytosis due to *Trichophyton verrucosum* detected from the clinical sample by SYBR-Green real-time PCR. The strategy was based on the DNA extraction directly from the infected hair followed by real‐time PCR and melting‐point analysis. A faster and differential diagnosis was observed when compared to the conventional mycological methodology for detection and identification of *Trichophyton verrucosum*.

## 1 Introduction

Dermatophytes are the most successful pathogenic fungi causing mycoses (dermatophytosis, also called ringworm) in superficial tissues, such as the epidermis, hair and nails. A wide variety of dermatophytes have been isolated from animals, namely *Microsporum canis*, *Microsporum gypseum* (*Nannizzia gypsea*), *Trichophyton mentagrophytes*, *Trichophyton equinum* and *Trichophyton verrucosum* [12].

*Trichophyton verrucosum* is the most common dermatophyte species involved in cattle ringworm, but also in other ruminants [4]. Dermatophytosis is often listed as self-limiting infection; however, animal dermatophytosis can spread between pets and livestock, as well as a zoonotic transmission to humans. Such human infections are usually highly inflammatory and result from the direct contact with cattle or infected fomites, involving the scalp, beard or other exposed areas of the body [6,8,13,15].

The diagnosis of dermatophytosis is based on clinical signs, mycological (confirmed by direct examination and identification in culture) and molecular methods [9]. Due to slow-growing of *Trichophyton verrucosum* and its atypical micromorphology features in culture compared to other dermatophytes [13], many laboratories only perform examination of hair (easy visualization of arthroconidia) in order to confirm dermatophytosis. The application of PCR and real-time PCR as diagnostic tools for dermatophytoses of hair, skin, and nail were recently highlighted [17]. Molecular and other methods are needed for faster and differential dermatological diagnosis and for the correct fungal identification, especially in cases the conventional methodologies (such as cytology and/or culture) do not allow the definitive diagnosis. Rapid diagnosis of the etiological agent is essential for effective epidemiological studies and implementation of antifungal treatment. The aim of this work was to report a bovine dermatophytosis case due to *Trichophyton verrucosum*. The detection was done by SYBR-Green real-time PCR using clinical sample directly.

## 2 Case presentation

A 2-year-old bovine was referred to the Veterinary Medical Teaching Hospital (HCV), Federal University of Rio Grande do Sul (UFRGS), Porto Alegre, RS, Brazil, presenting circular areas of alopecia covered with thin farinaceous desquamations and with thick crusty lamellar scales spread all over the body. No previous antifungal treatment was carried out. The diagnosis was conducted by direct observation under microscopy of fungal propagules (ectothrix arthoconidia visualization) in the hair and resuspension in potassium hydroxide. In addition, real-time PCR was applied for direct analysis of the fungal DNA obtained from the hair sample.

### 2.1 *Mycological diagnosis - Microscopic examination* of the hair

The sample was obtained by plucking the hair with forceps and scraping scales from the peripheral area of the lesions. Hair specimen was clarified and examined microscopically using 10% potassium hydroxide (KOH) for the visualization of chains of arthroconidia (ectothrix invasion of hair).

### 2.2 DNA extraction from hair sample

Hair sample was incubated overnight at 55 °C in 360 μL of lysis buffer and 20 μL QIAGEN protease (QIAamp DNA Mini kit, Qiagen, GmbH). Then, tungsten carbide beads were added to the previous mixture in a 2 mL tube. Disruption was performed with vortex at 1–2 min high‐speed (20–30 Hz). Subsequently, Qiagen DNeasy® plant mini DNA extraction kit protocol was used to extract DNA from the hair sample according to the manufacturer's instructions. DNA was eluted in 50 μL of elution buffer provided in the kit. Fungal DNA was frozen at −20 °C.

### 2.3 DNA extraction from dermatophytes (controls)

The reference strains (*Microsporum gypseum* ATCC 14683 and *M. canis* ATCC 11621) and one clinical dermatophyte isolate (*Trichophytom mentagrophytes*) were cultured on Mycosel Agar (BD®, Franklin Lakes, NJ, USA) at 25–30 °C for 10–15 days. A small piece of the dermatophyte was cut out from the agar and put in an eppendorf tube. The fungal DNA was extracted using QIAamp DNeasy Plant Mini kit (Qiagen) according to the manufacturer's instructions. DNA was eluted as mentioned above for hair sample.

### 2.4 Conventional PCR and sequencing

The clinical sample was submitted to conventional PCR using the same pan‐dermatophyte primers [2]. Polymerase chain reaction (PCR) amplification was performed a total volume of 25 μL containing 1 μL of DNA extract, 12.5 μL Qiagen Taq PCR master mix (Qiagen, Hilden, Germany) and 0.5 μL of each primer (for a 0.2 μM final concentration of each primer). The cycling parameters was as follow: pre-incubation at 94 °C for 15 min, 35 cycles of denaturation at 94 °C for 30 s, annealing at 60 °C for 90 s, extension at 72 °C for 1 min, and a final extension step of 10 min at 72 °C. Samples were analyzed by electrophoresis through 2% agarose gel (stained with ethidium bromide). PCR products were purified using PuriLink™ PCR Purification Kit (Invitrogen), and sequencing to confirm the identity. Geneious platform (Biomatters, Ltd., New Zealand) was used for sequence assembly, comparison and alignment.

### 2.5 SYBR green real time PCR identification (qPCR)

A real‐time PCR was performed using the pan‐dermatophyte primers for detecting a DNA fragment encoding chitin synthase 1 (CHS1), panDerm1 (5′- GAA GAA GAT TGT CGT TTG CAT CGT CTC -3′) and panDerm2 (5′- CTC GAG GTC AAA AGC ACG CCA GAG -3′) [2] using a StepOne™ Real-Time PCR System. The PCR mixtures contained 10 μL of SYBR Green PCR Mix (Applied Biosystems, Foster City, CA, USA), 0.5 μL of each primer (10 μM) and 2 μL of template DNA. H_2_O was added in order to achieve the reaction volume of 20 μL. Amplification was performed with the following cycling profile: 10 min of initial denaturation and enzyme activation step at 95 °C, followed by 40 cycles of 15 s for denaturation at 95 °C, and 1min for annealing/extension at 60 °C. The melting curve data were obtained by continuous fluorescence acquisition from 60 to 95 °C with a ramp rate of 0.3°. Total run‐time with melt curve analysis is 120 min.

### 2.6 Results

**Fig. 1:**
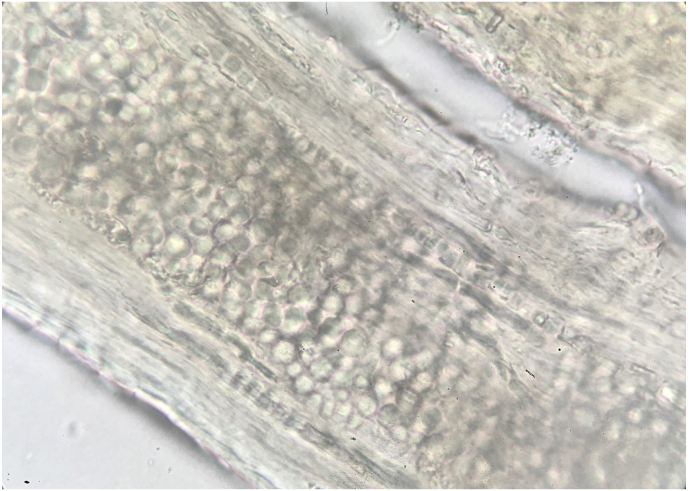
Infected hair with chains of ectothrix arthroconidia obtained from a cow with *Trichophyton verrucosum* (x400).

**Fig. 2:**
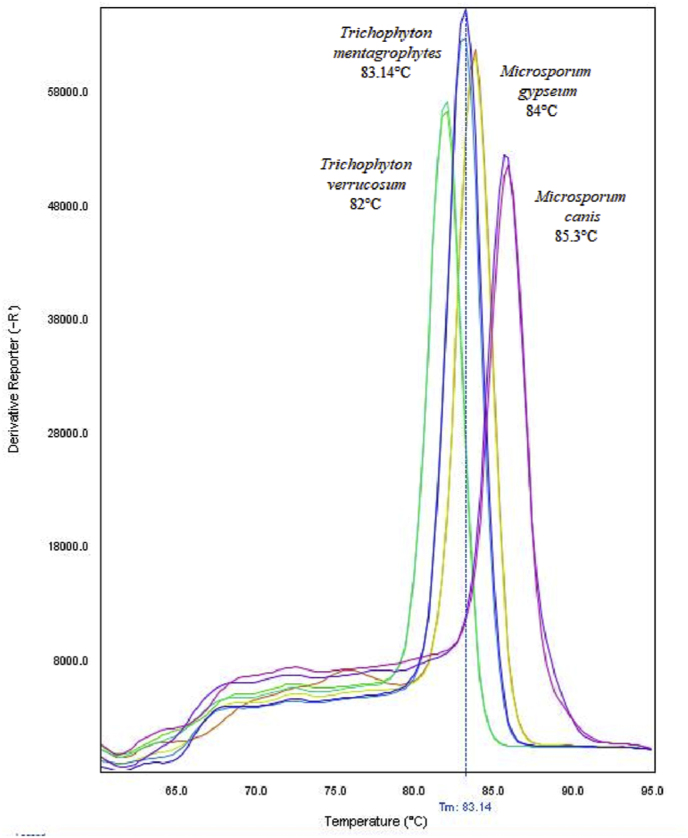
The different PCR products are separated by melting‐point analysis.

Microscopic examination of hair sample revealed extensive ectothrix invasion (Fig. 1). The hair sample and controls were positive in the conventional pan‐dermatophyte PCR. The dermatophyte present in the hair sample was confirmed as *Trichophyton verrucosum* following the amplification of CHS1 gene and sequencing analyses. The sequencing analyses revealed a CHS1 sequence with 100% similarity to the *Trichophyton verrucosum* chitin synthase gene available in the NCBI GeneBank (e.g. sequence ID EU363514.1). Real Time PCR showed it was possible to detect some dermatophytes and the specific PCR products could be separated successfully by conducting melting‐point analysis for multiple dermatophytes. The hair sample (*T. verrucosum)* melted at 82 °C, while *T. mentagrophytes*, *Microsporum gypseum* and *M. canis* melted at 83.14 °C, 84 °C and 85.3 °C respectively (Fig. 2), showing the identification of the clinical isolate was distinct from the references and other isolates used as controls.

## 3 Discussion

In veterinary dermatology, dermatophytosis is common and one of the most frequently involved fungal agents are *Microsporum canis*, *M. gypseum* and *T. mentagrophytes* considering all animal species [4,16]. Regarding bovine dermatophytosis, the main etiological agent is *T. verrucosum*. Its diagnosis is usually performed by direct examination, since its culture (reference standard for mycological diagnosis), requires specific incubation temperature (37 °C) and long incubation time (6–8 weeks) [1]. The direct examination of the hair sample allows a reliable screening test for initial dermatophytosis diagnosis [7,14], but does not allow the accurate diagnosis of the species. The size and disposition of arthroconidia differ depending on dermatophytes species: clusters of small (2–4 μm) arthroconidia for *M. canis*, (2–3 μm) arthroconidia for *T. mentagrophytes*, chains of various size conidia for *T. equinum* or chains of large (up to 12 μm) arthroconidia for *T. verrucosum* [4]. In our study, the direct examination of the hair was positive for the presence of arthroconidia and confirmed a case of bovine ringworm. Due to easy visualization of the arthroconidia, the dermatophyte species involved was suspected to be *T. verrucosum*, although additional tests were necessary to confirm the fungal identification.

There are several reports suggesting molecular identification for fungal cultures of *Microsporum* and *Trichophyton* spp. in veterinary medicine [3,5,11]. The number of reports applying molecular methods directly to human clinical samples of are still scarce, but these studies suggest satisfactory results and may soon be considered the standard reference for the diagnosis of dermatophytosis [10,11]. Conventional molecular methods confirmed the identification of this fungus as *T. verrucosum* using partial amplification and sequencing analysis of CHS1 gene.

The hair sample and reference strains were also positively amplified using real-time PCR and each specific product melts in a different temperature, ranging from 82 °C to 85.3 °C. The dermatophyte present in the hair sample had been confirmed as *T. verrucosum* by conventional PCR and sequencing and real-time PCR confirmed the identification. The diagnosis of dermatophytosis can be improved by complementary tests as wood's lamp test, histopathology, immunohistochemical (IHQ) analysis [9,14] and real-time PCR directly on clinical samples. It may be possible in the near future to use pan-dermatophyte primers as initial evaluations for diagnosis, relegating culture techniques as secondary and confirmatory tools in veterinary diagnostic laboratories.

The results from the microscopic examination (artroconidia ectotrix) of hair sample, conventional PCR/sequencing analysis and real-time PCR assay agreed on the final diagnosis of dermatophytosis for this case. The detection of dermatophytes directly from the clinical sample (hair) by real-time (qPCR) is a rapid diagnostic alternative, offering the detection, amplification and identification of fungal products in a few hours. In summary, this method based on DNA extraction directly from the hair and real‐time PCR melting‐point analysis give a faster and differential diagnosis when compared to the conventional mycological methodology, especially fungal culture. Further studies are necessary to confirm the feasibility of this technique in the routine diagnosis of dermatophytosis in veterinary laboratories.

## Ethical statement

Hereby, I Andréia Spanamberg consciously assure that for the manuscript **Bovine Ringworm - Detection of *Trichophyton verrucosum* by SYBR-Green real-time PCR**

The following is fulfilled.

1) This material is the authors' own original work, which has not been previously published elsewhere.
2) The paper is not currently being considered for publication elsewhere.
3) The paper reflects the authors' own research and analysis in a truthful and complete manner.
4) The paper properly credits the meaningful contributions of co-authors and co-researchers.
5) The results are appropriately placed in the context of prior and existing research.
6) All sources used are properly disclosed (correct citation).
7) All authors have been personally and actively involved in substantial work leading to the paper, and will take public responsibility for its content.

## Declaration of competing interest

There are none conflict of interests.
